# Relation between dimensional distinctiveness and comparison format in a novel noun generalization task in preschoolers

**DOI:** 10.3389/fpsyg.2024.1444287

**Published:** 2025-01-10

**Authors:** Yannick Lagarrigue, Jean-Pierre Thibaut

**Affiliations:** LEAD, CNRS, UMR 5022, Université Bourgogne Europe, Dijon, France

**Keywords:** novel noun generalization, shape bias, comparison, dimensional distinctiveness, development

## Abstract

Numerous studies have shown that in novel noun generalization tasks, the simultaneous presentation of multiple learning examples increases the percentage of generalizations that are based on *a priori* less salient properties, compared to the presentation of a single learning example. In this research with preschoolers (*n* = 300) we demonstrate that this effect can be modulated by dimensional distinctiveness, i.e., how easy it is to determine whether two dimension values (shape and 2D texture) are easy to distinguish or not. In a first experiment, we manipulate dimensional distinctiveness globally (both shape and 2D texture are distinctive, or not) and explore how it interacts with comparisons format: two learning examples from the same category (i.e., within-category comparison), two learning examples from different categories (i.e., between-category comparison), and no-comparison (i.e., only one learning example). Results show that within-category comparisons yielded more taxonomic generalizations than between-category comparisons and no-comparison conditions. Furthermore, children selected more often the taxonomic match with highly distinctive stimuli than with low distinctive stimuli. In a second experiment, we independently manipulate the distinctiveness of stimuli shape and 2D texture to determine which dimension distinctiveness might contribute to better generalization in a within-comparison format. Results indicated that within-category comparisons resulted in more taxonomic generalization with distinctive textures, regardless of shape distinctiveness. These findings suggest that not all comparison conditions are equals and that children’s generalizations may be influenced by the characteristics of the stimuli.

## 1 Introduction

The capacity of young children to establish the reference of novel words with minimal exposure to objects is truly remarkable. Understanding how they make sense of the objects dimensions and interpret novel word reference is a crucial question for developmental science. In the real world this means understanding how children know which dimensions characterize entities that, for example, are named *dogs* or *tables* and which properties are not relevant. One standard method is the novel word extension task in which children are taught a novel word that is illustrated with one (or more) instance(s). Then they are shown new referents and asked which one(s) could also share the same name (Bloom, 2002; Gelman, 2003; Gentner and Namy, 1999; Imai et al., 1994; Landau et al., 1988; Markman, 1989; Waxman and Leddon, 2011, among many others).

In the present study, we focus on a learning design, the comparison design, which has been shown to facilitate category learning and novel word generalization when salient similarities (e.g., such as shape) lack conceptual relevance while the similarities unifying the category (e.g., texture similarities) are *a priori* less salient (Alfieri et al., 2013; Augier and Thibaut, 2013; Gentner and Namy, 1999; Namy and Clepper, 2010; Price and Sandhofer, 2021; Stansbury et al., 2024; Thibaut and Witt, 2023; Twomey et al., 2014). Indeed, a large body of recent studies shows that comparing two or more learning stimuli leads to more conceptually based generalizations [e.g., taxonomically-based versus perceptually-based], of novel words than single stimulus learning condition (see Imai et al., 1994). In the present contribution, we focus on object nouns.

Decades of research on lexical learning show that very young children are able to map words to objects with very few presentations of the word, even a single exemplar (Carey, 2010; Carey and Bartlett, 1978; Horst and Samuelson, 2008; Landau, 1994; Landau et al., 1988; Markman, 1990; Markson and Bloom, 1997; Waxman, 1990; Woodward et al., 1994). The single object design has been extensively used in the study of novel words especially the extension of novel object nouns and what authors have described as lexical biases, that is the set of hypotheses young children consider when they have to extend novel words (Imai and Haryu, 2004; Markman, 1989, 1990; Markman and Hutchinson, 1984; Waxman, 1990).

For example, the shape bias (Baldwin, 1992; Diesendruck and Bloom, 2003; Graham and Poulin-Dubois, 1999; Imai et al., 1994; Landau et al., 1988; Poulin-Dubois et al., 1999; Tek et al., 2012) posits that young children generalize novel object nouns to objects sharing the same shape rather than the same size, texture, or color as shown in a seminal paper by Landau et al. (1988). This bias is language specific and disappears when no noun is provided (Gentner and Namy, 1999; Graham et al., 2010). It has received several interpretations, that is a learned bias for Smith et al. (2002), or the result of the appeal of shape similarities for Imai et al. (1994). This shape bias is most often conceptually relevant as, hammer-shaped objects are the best candidates to fulfill the function of hammers and, hence, be *hammers*. However, as argued by Imai et al. (1994), there are cases in which shape lacks conceptual relevance (e.g., tennis balls will never been called *apples*, except in non-literal language) and other properties, less salient, are conceptually relevant (e.g., laying eggs or hollow bones for birds). In this work, we focus on situations in which shape is not the unifying dimension and investigate which conditions might prompt noun generalizations based on less salient properties.

An increasing number of studies in novel word generalization task demonstrates that providing children with an opportunity to compare several exemplars (at least two) favor taxonomic generalizations more than single example presentations do. In one pioneering study, Gentner and Namy (1999) used pictures of familiar categories (e.g., fruits, animals or vehicles) and tested four-year-olds in a novel-name generalization task. Children were assigned to one of two experimental conditions. In the comparison condition, two learning examples were introduced simultaneously with the same pseudo-noun. For example, an apple and a pear were pointed at as “blickets” (“This is a blicket” and “This is a blicket too”). In the no-comparison condition, only one example was presented as a “blicket”. In both generalization conditions, children were asked to choose between two options, a perceptually similar object (e.g., a balloon) and a taxonomically related but perceptually dissimilar object (e.g., a banana), which one was also a “blicket.” In the no-comparison condition, the majority of children generalized the novel word to the perceptually similar object (i.e., the balloon). Importantly, this pattern was reversed in the comparison condition, in which a majority of children selected the taxonomically related option (i.e., the banana). Subsequent studies have consistently supported this initial result (Graham et al., 2010; Hammer et al., 2009; Namy et al., 2007; Namy and Clepper, 2010; Namy and Gentner, 2002; Price and Sandhofer, 2021; Stansbury et al., 2024), exploring various linguistic categories such as names for parts (Namy et al., 2007), adjectives (Mintz, 2005; Mintz and Gleitman, 2002; Waxman and Klibanoff, 2000), action verbs (Childers, 2011, 2020; Childers et al., 2014, 2016), relational nouns (Gentner et al., 2011; Kurtz and Boukrina, 2004; Thibaut and Witt, 2015, 2023) or labels for spatial relations (Christie and Gentner, 2010).

How do comparisons facilitate conceptualization? Gentner and Namy (1999) argue that, in contrast with single stimuli formats, comparisons lead to a deeper, more conceptually-based encoding of the stimuli properties such as relations or non-salient perceptual properties (e.g., Gentner and Namy, 1999; Goldstone et al., 2010; Hammer et al., 2009; Namy and Gentner, 2002). Namy and Gentner (2002) posit that common labels *invite the comparison*. This promotes the alignment of the compared stimuli in terms of their constitutive properties. Salient perceptual similarities are first aligned. Then, further comparisons lead to the progressive emergence, discovery, of deeper, less immediate, similarities until a maximally consistent match between stimuli is reached. In this model, salient similarities are the starting point on which children build deeper, less obvious, similarities.

However, beyond this general description of the effects of comparison on word generalization, previous studies have shown that several factors modulate its “generalization” efficiency. First, the presence of a noun (rather than its absence) makes comparison more effective (Gentner and Namy, 1999; Graham et al., 2010; Namy and Gentner, 2002). According to many authors (Booth and Waxman, 2009; Gentner and Namy, 1999; Graham et al., 2010; Mintz and Gleitman, 2002; Namy and Gentner, 2002), the presence of a noun elicits the search for deep commonalities in the comparison process, not comparison itself. We also know that the semantic distance between learning items and the number of learning stimuli matters (Gentner and Namy, 1999; Thibaut and Witt, 2015, 2023). For example, Thibaut and Witt (2023) manipulated the semantic distance between learning items by presenting either pairs of taxonomically close items (e.g., a red apple and a green apple) or more distantly related pairs (e.g., a red apple and a cherry). They defined semantic distance in terms of levels in the taxonomic hierarchy. They also manipulated the distance between the learning items and the taxonomically related target (e.g., a same immediate superordinate category target like fruit, or a more distant superordinate level category like meat). They have shown with four-and six-year-old children that generalization performance was higher when learning items were from the same superordinate level category rather than from the same basic level category. Augier and Thibaut (2013) and Thibaut and Witt (2015, 2023) interpreted similar results in terms of executive functions, highlighting the need to monitor information and manage comparisons in the comparison conditions.

The present paper expands on former studies, investigating two related variables that might also modulate the output of stimulus comparisons. Our central hypothesis was that the structure of the learning stimuli, that was implemented here as stimulus distinctiveness, might influence novel noun generalization in comparison and no-comparison designs. Distinctiveness would influence the way children parse stimuli into their components and, thus, novel noun learning and generalization. Here, we define distinctiveness in terms of the differences between stimuli. It refers to the ease with which they can be distinguished (see materials) when stimuli are defined around the *same set of* dimensions. As Hammer and Diesendruck (2005) put it “The physical differences between stimuli, that is, their distinctiveness” (p. 138). The authors showed that distinctiveness also “affect people’s (…) ability to discriminate between stimuli or to perceive them as similar” (p. 138). If two stimuli are defined along the same set of dimensions (e.g., color, texture, shape) in a multidimensional space, the closer they are on each dimension, the less distinctive they are. Distinctiveness can be captured by a measure of the stimuli’s overall distance in a multidimensional space (Engelthaler and Hills, 2017; Nosofsky, 1986). Note that no commitment to any theory about the nature (e.g., format) of these dimensions is implied by this definition. This measure of similarity differs from other measures of similarity relying on the number of common features and the number of features that contrast them (Goldstone and Son, 2012; Murphy, 2002; Tversky, 1977).

We hypothesize that distinctiveness influences generalization because previous studies have shown that young children (preschoolers) perceive less distinctive stimuli as holistically more similar one to the other and, as a result, are more difficult to parse into their dimensions (e.g., Shepp et al., 1987; Smith, 1989; Smith and Kemler, 1978). These studies suggest that less distinctive objects are more difficult to perceive as similar or different objects, or in other words, how easy it is to detect on which dimensions they are similar or different. Available evidence suggests that distinctiveness might affect novel noun generalization because when dimension distinctiveness is low, demands on memory and attention increases and young children have greater difficulty extending a label (see Tek et al., 2012). Along a similar line, Cimpian and Markman (2005) argued that most studies on novel noun learning have used simple shapes which could have enhanced the salience of shape. They showed with more complex shapes that the rate of shape-based answers decreased in favor of taxonomic choices (i.e., a smaller shape bias). Even though the authors used a single design, they showed that the structure of the stimuli (simple or complex) influenced word extension. In our study we test whether the structure of the stimuli, in terms of distinctiveness, would influence the role shape plays and might interact with comparison formats in a novel noun generalization task. As far as we know, no study has tested whether distinctiveness would influence how stimuli are analyzed then aligned in a comparison design and how this factor would influence novel noun generalization. While previous studies, have already explored how feature distinctiveness and salience affect novel word generalization in children, these investigations were not conducted in the framework of direct comparison. The present study builds upon early works by examining how the comparison process itself may alter children’s handling of feature distinctiveness. We posit that both distinctiveness and comparison change the way children attend to and prioritize specific features of stimuli in a novel word generalization situation.

If distinctiveness refers to the “internal structure” of the stimuli (see above), this factor might interact with a related factor that we can isolate as the category status of the information itself. Here, category status refers to the categories the learning stimuli belong to, same category or different categories. Formally, authors distinguish what they call within-category comparisons from between-category comparisons. In within-category comparisons, the exemplars are introduced with the same noun (“This is a dax and this is also a dax”). In between-category comparisons, the first exemplar is introduced with a given noun and the second exemplar is introduced as not sharing the same noun (“This one is a dax but this one is not a dax” or “This one is a dax and this one is a dajo”).

Within-category comparisons reinforce a shared category membership, which may encourage children to focus on common properties across exemplars. As Hammer et al. (2008) explain, within-category comparisons emphasize commonalities (i.e., all daxes have the property i) and decrease the importance of properties contrasting the stimuli with the same noun (i.e., *dax* 1 has feature j, *dax* 2 not, thus feature j does not characterize *daxes*). On the contrary, between-category comparisons highlight differences, potentially directing children’s attention toward distinctive properties. Hammer et al. (2008) suggested that shared feature(s) between categories (i.e., the *dax* and the non-*dax* have property k in common, so k is not a diagnostic property for daxes) are less informative because they fail to discriminate between categories. Thus, features shared by categories X and Y are not very informative, as they do not discriminate them and provide no clue as to the features that distinguish them and, more crucially, maybe, do not provide any information regarding unifying features, within the category. We acknowledge that these labeling conventions might not only reflect category membership but also influence the salience of specific dimensions (e.g., shape or texture). For instance, introducing different labels in between-category comparisons might shift children’s attention toward unique aspects of each exemplar rather than shared characteristics.

**Figure 1:**
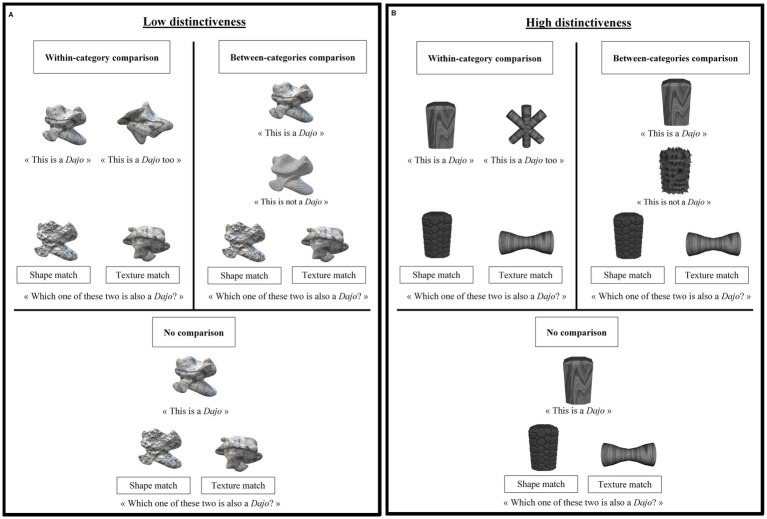
**(A)** Example of stimuli and instructions used in the three comparison conditions of the low distinctiveness condition. **(B)** Example of stimuli and instructions used in the three comparison conditions of the high distinctiveness condition.

The evidence regarding cumulated within and between (contrast) category remains scarce and inconclusive. With familiar items, Namy and Clepper (2010) compared a within-category condition with a within-plus a between-category comparison (e.g., a bicycle introduced as a *dax* and a stimulus from another category, e.g., barbells as *not a dax*). The task was similar to Gentner and Namy (1999) and the authors found no additional effect of the introduction of the between-category comparison, that is not more taxonomically related choices. Closer to us, with unfamiliar stimuli similar to the ones we are using here (see Figure 1), Augier and Thibaut (2013) contrasted within and between-category conditions. They assessed which conditions would promote generalization based on a unifying low saliency dimension (2D texture in their paper) rather than on a high saliency but non-unifying dimension such as shape. A contrast category condition (that is “A is a dax” but “B is not a Dax” condition) did not increase the number of 2D texture choices compared to a single object condition, whereas a within-category condition (“both are daxes”) increased the number of texture choices in both 4-and 6-year-olds. However, when both sources of evidence were introduced (this is a “dax” and this is also a “dax,” but this one is not a “dax”), younger children (4-year-olds) performance was lower than when only the within-category stimuli (“both are daxes”) were available. For six-year-olds, adding contrast (between-category) stimuli slightly increased the number of selections of the less salient dimension (2D texture), which indicated an interaction between comparison conditions and age. Price and Sandhofer (2021) also used unfamiliar objects and confronted a single stimulus condition with a within comparison (three same-shape objects) and a contrast condition with three different-shape objects. Surprisingly, the single condition led to better generalization than the two comparison conditions. However, in the three conditions, the expected generalization stimulus was a same shape object, that is a match on the salient dimension. Overall, this brief overview shows that the evidence regarding within-versus between-category conditions remains scarce and inconclusive.

We investigate whether variations in dimension distinctiveness influence performance in comparison formats (within-and between-category) in contrast with no-comparison formats, with 3-to 6-year-old children. The no-comparison (single object) condition was our baseline and, given former results, was supposed to elicit shape-based answers. Our main hypothesis was that object distinctiveness influences the discovery of the features for generalization (Namy and Gentner, 2002; Schyns et al., 1998). In two experiments, we used unfamiliar stimuli, similar to the ones used by Augier and Thibaut (2013) varying in shape and 2D texture and capitalized on the *a priori* saliency of their shape in novel names generalizations (Kucker et al., 2019). With 2D images, Augier and Thibaut (2013), Graham et al. (2010), and Hartley and Whiteley (2024) confirm that the texture they used is a less salient dimension than shape: in a no-comparison condition, children chose same-shape stimuli, because of the a priori shape saliency. The key point is that the 2D implementation of texture was a priori less salient than the implementation of shape. Other features, like size (Gelman and Meyer, 2011; Landau et al., 1988), were a priori less salient dimensions compared to shape. To the best of our knowledge, no study has manipulated distinctiveness in a novel name learning task with a comparison design and considered possible interactions with (no-) comparison formats. Our aim was to examine children’s generalization of novel nouns when a salient feature like shape does not unify the displayed stimuli. Shape and 2D texture were used here for two reasons: (1) shape is a priori more salient than 2D texture in a novel noun learning task. (2) We could manipulate their distinctiveness separately. Our aim was to examine children’s generalization of novel nouns when a salient feature such as shape does not unify the displayed stimuli. By using 2D textures, we aimed to create a “non-default” case that could reveal how children manage less salient categorical features. This approach sheds light on how children adapt to and prioritize features beyond the more commonly usual shape cues, in line with feature distinctiveness within a comparison framework.

In the first experiment, we manipulated the global stimulus distinctiveness and the status of the compared stimuli (within-or between-category). In the second experiment, we only kept one comparison condition (the within-category comparison, see below), but systematically studied the contribution of each dimension distinctiveness.

## 2 Experiment 1

In the first experiment, we contrasted high-distinctiveness stimuli built around a highly distinctive shape and a highly distinctive 2D texture with low-distinctiveness stimuli built around a low distinctive shape and a low distinctive 2D texture. We also contrasted the format of the stimuli presentation, contrasting within-category, between-category comparisons and no-comparison conditions. The third purpose was to study potential interactions between the internal structure of the stimuli (distinctiveness) and the structure of the stimuli display (types of comparisons). We studied three-to-six-year-old children given that most previous studies involved similar ages which were highly sensitive to experimental variations (Augier and Thibaut, 2013; Graham et al., 2010; Namy and Clepper, 2010). Children younger than 3 years of age had difficulties with the comparison paradigm and, beyond 6 years old, the generalization task might be too easy.

Our hypotheses were that the no-comparison condition should lead to a majority of shape choices, especially in the high-distinctiveness condition in which differences in shape will be easily detected, leading to the same-shape option. In contrast, less distinctive stimuli should elicit more inconsistent profiles (participants choosing more randomly between the same shape and same 2D texture options).

Second, former studies predict that high-distinctiveness stimuli should lead to more shape-based choices than low-distinctiveness stimuli since a more distinctive shape should attract more attention and alignments on the base of shape than a less distinctive shape. However, the opposite hypothesis might also be true, that is more 2D texture-based choices in the high-distinctiveness condition. This is because highly distinctive shapes might be easier to reject as irrelevant because it is easy to notice that they do not unify the two learning stimuli, whereas highly distinctive textures are easier to notice as unifying and, thus, stimuli are easy to align on this dimension. Low-distinctiveness stimuli should be perceived more holistically (Deng and Sloutsky, 2015; Smith and Kemler, 1978) and elicit more random choices than in the highly distinctive case.

Third, comparison format (i.e., within and between) should interact with distinctiveness. One possibility is that differences between within and between-category comparison might be smaller in the low-distinctiveness case than in the high-distinctiveness case because children might be more inconsistent due to their difficulties parsing the stimuli into their dimensions.

### 2.1 Method

#### 2.1.1 Participants

Three hundred (300) preschoolers from three to 6 years old participated in this study and were tested individually in a quiet room at their school. They were predominantly Caucasian and came from middle-class urban areas. The schools were mainly located in the center of the city and areas around, which are middle class and upper middle class (for socio-demographic information regarding the city see https://www.insee.fr/fr/statistiques/2011101?geo=COM-21231). Participants were randomly assigned to one of the six experimental conditions with 50 children per condition (Table 1). Previous experiment (Thibaut and Witt, 2023) manipulating information young children deal with during comparisons used groups of 33 to 34 children per group, suggesting that our sample size (*n* = 50 per group) enable us to test our hypotheses.

**Table 1:** Participants’ age and gender for each condition of Experiment 1.

	Conditions						Comparison
Condition of comparison	Within-category		Between-category		No-comparison		—
Distinctiveness	High	Low	High	Low	High	Low	—
Number of participants	50	50	50	50	50	50	—
Age (in months)	60.75 ± 8.10	60.30 ± 7.30	59.48 ± 9.00	58.93 ± 8.60	59.32 ± 9.30	60.02 ± 7.79	*F* (5,294) = 0.32, *p* = 0.90
Gender (F/M)	22/28	25/25	21/29	26/24	26/24	20/30	—

F, females; M, males.

All experiments followed institutional ethics board guidelines for human research. This study was ethically approved by an official agreement (Convention No. 2019-0679) between the Academia Inspection of the French National Education Ministry, the University of Bourgogne (“Inspection Académique de Côte d’Or”), and our laboratory. The official agreement between the laboratory, the university and the Academic Inspectorate enshrines the 7 principles of the French Society of Psychology’s (SFP) ethical code: right to research and knowledge, investigators’ and research director’s qualifications, investigators’ and research director’s responsibilities, respect of participants’ rights and right to withdraw from the study, privacy, free and enlightened consent by participants, use and publication of results. The agreement also ensured that the schools’ regulations regarding children’s rights were followed and that complete anonymity was ensured for all participants. An information letter was sent to parents who, then, returned their written consent.

#### 2.1.2 Materials

Seven sets of five artificial 2D grey-scale objects were created for each of the two levels of distinctiveness, low and high (i.e., a total of 14 sets) and printed on laminated cards. The sets were adapted from Augier and Thibaut (2013). Each set was composed of three examples, two from the same category (for the within-category comparison condition) and one from another category (for between-category comparison condition), and two test objects (see Figure 1). The two standards always had the same 2D texture but different shapes. The contrast object had the same shape as one of the two standards but differed from both standards on texture. The first test object, the shape match, had the same shape as one of the two standards but a different texture. The second test object, the texture match, had the same texture as both standards but a different shape (see Figure 1).

Each object was printed on a 12 cm by 9 cm laminated card. The objects’ 2D textures and shapes in the low-distinctiveness condition were created to be less distinctive (Figure 1A) than in the high-distinctiveness condition (Figure 1B). To assess whether low-distinctiveness stimuli were less distinctive than highly distinctive stimuli we asked 75 adults to rate the perceptual distinctiveness of pairs of objects on a 7-point Likert-like scale (ranging from not distinctive at all to extremely distinctive). Two ratings were carried out, one for 2D texture distinctiveness, one for shape distinctiveness. Each possible pairs of objects crossing the seven sets of each condition were presented in a pseudo-randomized order between participants. For each pair of objects participants were either presented with two objects with the same shape and asked to judge 2D texture distinctiveness or presented with two objects with the same 2D texture and asked to judge shape distinctiveness (see Table 2 for results). On average, the pairs of objects were judged to have significantly less distinctive 2D texture in the low-distinctiveness set (mean = 1.67, SD = 1.19) than in the high-distinctiveness set [mean = 3.47, SD = 1.19, *t* (74) = 15.85, *p* < 0.001]. On average, the pairs of objects were also judged to have significantly less distinctive shape in the low-distinctive set (mean = 1.81, SD = 1.10) than in the high-distinctiveness set [mean = 3.48, SD = 1.29, *t* (74) = 13.82, *p* < 0.001]. While this method is often used in studies with children and offers a reliable baseline, adults’ ratings may not fully capture how preschool-aged children perceive these differences rather distinctions.

**Table 2:** Means (of scores from 0 to 7), standard deviations and confidence intervals of judgments for all conditions of distinctiveness.

	High-shape/low-texture	High-shape/high-texture	Low-shape/high-texture	Low-shape/low-texture
Mean score for shape ± standard deviation Confidence interval	3.87 ± 1.22 3.51–4.24	3.48 ± 1.29 3.19–3.77	1.35 ± 0.85 1.10–1.61	1.81 ± 1.10 1.56–2.06
Mean score for texture ± standard deviation Confidence interval	1.47 ± 1.15 1.12–1.81	3.47 ± 1.19 3.20–3.74	3.84 ± 1.12 3.50–4.17	1.67 ± 1.19 1.40–1.94

We also used 14 different bi-syllabic labels (pseudo-words), from Augier and Thibaut (2013). Bi-syllabic pseudo-words are, as shown by Gathercole and Baddeley (1993), easier to remember than monosyllabic pseudo-words. Each of these novel nouns (Youma, Buxi, Dajo, Zatu, Sepon, Xanto, Vira, Loupo, Sampi, Loga, Kufa, Joru, Rodon, and Budan) was randomly associated with one of the 14 sets. The names were pseudo-randomized in a counterbalanced design across sets and participants.

#### 2.1.3 Procedure

The experimental paradigm was the same novel noun generalization task as in former studies (Augier and Thibaut, 2013; Gentner and Namy, 1999) in which children have to decide which of two simultaneously presented test objects have the same noun as the example(s). Each child performed seven trials. In each trial, children were introduced to a puppet called “Yoshi” who “lives very very far away” and were told that Yoshi had some unknown objects with strange names that they would have to learn. The experimenter introduced the first example with a novel noun (e.g., “This is a *dajo*”) and children were asked to repeat the novel word (see the list of the pseudo-words above). In the within-category comparison conditions, the second example was introduced with the same label as the first one (e.g., “Look, this is a *dajo* too”). The two examples were presented in a row and their location (right or left) was determined randomly. In the between-category comparison situations, the second example was introduced as a non-member of the category (e.g., “This is not a *dajo*”). In all the trials, the two learning examples were presented sequentially and left in view during the entire trial. Children were asked to look carefully at the objects. Then, the two test objects (i.e., the shape and the texture matches) were introduced simultaneously and children were asked to point to the one with the same name as the two standards (e.g., “Show me which one of these two is also a *dajo*”). The test objects were presented in a row and their location was determined pseudo-randomly in a counterbalanced design.

The first two trials were training trials. The two training sets were picked randomly among all the sets of objects. The experimenter checked that children understood the game by asking them whether the test object that was not selected by the child could also go with the standard(s). In case of a positive answer, the experimenter explained that only one of the test objects could go with the standards. During these training trials, some children picked one of the standards so the experimenter had to repeat that the two standards could be called with the same name (e.g., “This one is a *dajo* and this one too”), and that he/she has to look for another one that might also be called *dajo*. These irrelevant responses were very rare after these two trials. No feedback was given, either during practice or training trials, in order to avoid biasing children’s subsequent responses.

#### 2.1.4 Data analysis

For each participant, we calculated the percentage of 2D texture match choices. The first two trials were training trials and were excluded from the analysis. The data came from the five following trials. Thus, the scores ranged from 0 to 100%, with increments of 20%. Then, we computed the mean percentage of 2D texture match choices of all participants in each of the six conditions. Note that, in the present context as in similar studies, texture refers to a 2D implementation of texture (see above). Hence, here after, texture refers to this 2D implementation.

#### 2.1.5 Statistical analysis

The design was a 3 × 2 ANCOVA with comparison (within-category vs. between-category vs. no-comparison) and distinctiveness (low-distinctiveness vs. high-distinctiveness) as between-subjects factors and age as a covariate.

Prior to the analyses, we ran an ANOVA with the six experimental conditions as between-subject factors on the mean age of children to ensure that mean age of children did not differ between the conditions (see Table 1).

We estimated the Bayes factors for these data using Jamovi (Jamovi Project^©^) and referred to the decision categories defined by Jeffreys (1998) and Kass and Raftery (1995). The Bayes factor, BF_10_, measures the likelihood of H_0_ vs. H_1_ given our data. We used the standard non-informative Cauchy prior in Jamovi (module imported from JASP, 33), with a default width of 0.707.

We also reported 95% confidence intervals. A significance level of 0.05 was adopted for all analyses. All results are plotted using means and standard errors.

### 2.2 Results

#### 2.2.1 ANCOVA analysis

To assess the effects of comparison, distinctiveness, and of their interactions, we conducted a two-way ANCOVA with comparison (within-category vs. between-category vs. no-comparison) and distinctiveness (high vs. low-distinctiveness) as between-subject factors and age as a covariate on the percentage of texture match choices. When appropriate, the data were further analyzed with post-hoc analysis (Tukey HSD) and the mean percentage of texture-based responses in each condition was compared to chance (50%) with a one sample *t*-test.

The ANCOVA revealed a significant main effect of comparison (see) [*F* (2, 293) = 47.76, *p* < 0.001, *η*^2^_P_ = 0.25, BF_10_ >1,000, which corresponds to decisive evidence in favor of H_1_]. Post-hoc analyses revealed that the percentage of texture-based responses were higher in the within-category comparison (M = 61.20%, CI = 54.25–68.15) condition than in the between-category comparison condition (M = 25.20%, CI = 18.78–31.62; *p* < 0.001, *d* = 1.27), and in the no-comparison condition (M = 20.20%, CI = 14.19–26.21; *p* < 0.001, *d* = 1.11). However, the post-hoc did not reveal differences between the no-comparison condition and the between-category condition (*p* = 0.52, *d* = 0.16).

There was no significant effect of distinctiveness [*F* (1, 293) = 2.84, *p* = 0.09, *η*^2^_P_ = 0.01, BF_10_ = 16.88, which corresponds to strong evidence in favor of H_1_] and of age [*F* (1, 293) = 0.13, *p* = 0.723, *η*^2^_P_ = 0.00, BF_10_ = 0.14, which corresponds to substantial evidence in favor of H_0_].

**Figure 2:**
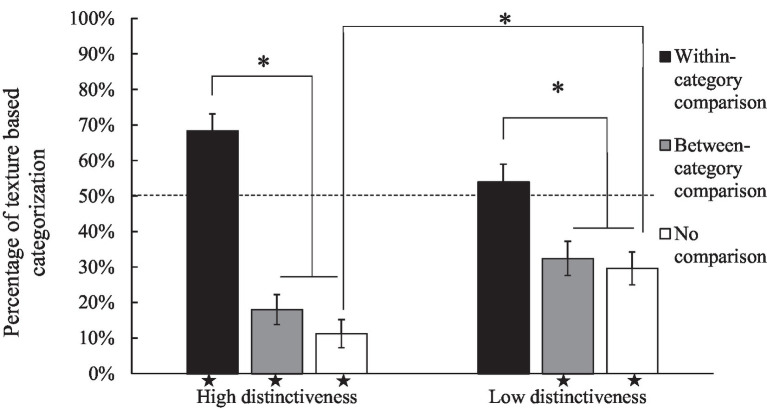
Mean percentage of texture-based categorization as a function of the condition of comparison and distinctiveness. A star at the bottom of a bar means that the corresponding condition significantly differed from chance (50%). Vertical bars represent standard errors of the mean. ^*^p < 0.05.

The most important result was the significant interaction between comparison and distinctiveness [*F* (2, 293) = 7.79, *p* < 0.001, *η*^2^_P_ = 0.05, BF_10_ = 68.23, which corresponds to very strong evidence in favor of H_1_]. Post-hoc analyses revealed that the only significant difference between the two levels of distinctiveness was in the no-comparison condition (see Table 3), in which the low-distinctiveness condition (M = 29.60%, CI = 20.42–38.78) gave more texture-based responses (see Figure 2) than the high-distinctiveness condition (M = 10.80%, CI = 3.88–17.72). Moreover, in both distinctiveness levels, the percentage of texture choices in the within-category comparison conditions (high: M = 68.40%, CI = 58.96–77.84, low: M = 54.00%, CI = 44.09–63.91) was significantly higher than in the no-comparison conditions (high: M = 10.80%, CI = 3.88–17.72; low: M = 29.60%, CI = 20.42–38.78, see Table 3) and in the between-category comparison condition (high: M = 18.00%, CI = 9.75–26.25; low: M = 32.40%, CI = 22.90–41.90, see Table 3). Overall, these results show that within-category comparison led to more texture-based answers than between-category comparison.

**Table 3:** Post-hoc of the interaction between comparison and distinctiveness on the percentage of texture-based responses.

		Within-category		Between-category		No-comparison	
		High	Low	High	Low	High	Low
Within-category	High		*p* = 0.22 *d* = 0.45	***p* < 0.01** ***d* = 1.57**		***p* < 0.01** ***d* = 1.79**	
	Low				***p* = 0.01** ***d* = 0.67**		***p* < 0.01** ***d* = 0.76**
Between-categories	High				*p* = 0.22 *d* = 0.45	*p* = 0.87 *d* = 0.22	
	Low						*p* = 1.00 *d* = 0.09
No-comparison	High						***p* = 0.04** ***d* = 0.58**
	Low						

Significant comparisons (*p* < 0.01) are reported in bold.

#### 2.2.2 Comparison with chance (50%)

Comparisons to chance are interesting as they reveal (or not) a bias towards one of the two options. In each of the six experimental conditions, we conducted a comparison of the percentage of texture-based responses against chance (50%) using a one-sample *t*-test. The results revealed distinctive patterns across conditions. In the no-comparison conditions children consistently exhibited a preference for shape-matches over texture-matches, as indicated by significantly lower percentages of texture-based responses (high-distinctiveness: *p* < 0.001, *t* = −11.1, *d* = −1.57; low-distinctiveness: *p* < 0.001, *t* = −4.35, *d* = −0.61). Similarly, in the between-category comparison conditions, children, regardless of distinctiveness levels, showed a significant preference for shape-matches over texture-matches (high-distinctiveness: *p* < 0.001, *t* = −7.60, *d* = 1.08; low-distinctiveness: *p* < 0.001, *t* = −3.63, *d* = 0.51). In the within-category comparison conditions, the percentage of texture-based responses differed significantly from chance only in the high-distinctiveness condition (*p* < 0.001, *t* = 3.82, *d* = 0.54), not in the low-distinctive condition (*p* = 0.433, *t* = 0.79, *d* = 0.11).

In summary, these results consistently demonstrate that in the absence of comparison and in the between-category comparison conditions, children tended to favor shape-match over texture-match. Within-category comparisons revealed a larger preference for our 2D implementation of texture, particularly in the high-distinctiveness condition.

#### 2.2.3 Consistency patterns analysis

Following Augier and Thibaut (2013), we also analyzed individual consistency patterns of answers in order to have a clearer view on participants’ consistency of responses across the five trials.

**Figure 3:**
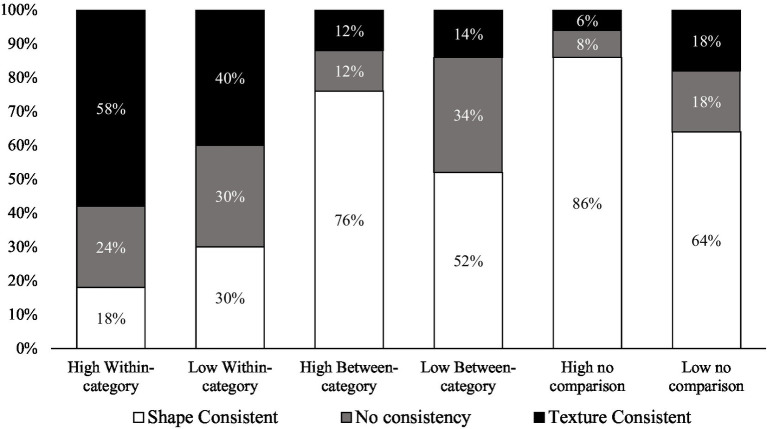
Percentage of children who were either texture-consistent, shape-consistent, or inconsistent for each experimental condition. “High” stands for high distinctiveness, and “low” stands for low distinctiveness.

Indeed, mean percentages of responses do not always do justice to the variety of the underlying response patterns, which provide important insights into the children’s decision-making. For instance, selecting texture 50% of the times is compatible with very distinct responses profiles: a bimodal pattern (in which some children predominantly select shape, while others predominantly select texture) or a unimodal pattern around 50%, or a scattered distribution along the entire performance scale. Thus, the purpose of this analysis was to clarify whether children were making consistent choices, based on a specific feature (either shape or texture) or were answering randomly. Consistency patterns provide information about distributions of responses which go beyond averages. We classified children into three groups, texture-consistent when they chose at least four texture matches out of five experimental trials, shape-consistent when they chose at least four times the shape matches out of five, and inconsistent in other cases. Pearson’s chi-square tests of independence were then run-on patterns of consistency (see Figure 3).

Analyses of individual consistency patterns in responses confirmed the above analyses. We examined (1) whether, within a specific comparison condition, the distribution of profiles varied between the two distinctiveness levels, and (2) whether, within a given distinctiveness condition, the distribution of profiles differed between the types of comparison.

1. In the no-comparison condition there were more shape consistent profiles in the high-distinctiveness than in the low-distinctiveness condition [*χ*^2^ (2, 100) = 6.54, *p* = 0.038]. This suggests that high-distinctiveness objects mostly elicited shape-based selections. In the between-category comparison condition there were also more shape-consistent profiles and fewer inconsistent profiles in the high-distinctiveness condition than in the low-distinctiveness condition [*χ*^2^ (2, 100) = 7.59, *p* = 0.023]. This suggests, again, that low-distinctive objects lead to more choice uncertainty. In the within-category comparison condition, there was no difference in terms of profile distribution between the two distinctiveness levels, with, in both conditions, more texture-based choices than shape-based choices [*χ*^2^ (2, 100) = 3.49, *p* = 0.018]. This suggests that children benefit from within-category comparisons, irrespective of the distinctiveness condition.
2. In the *low* distinctiveness condition there were more shape consistent and more inconsistent profiles in the no-comparison [*χ*^2^ (2, 100) = 11.82, *p* = 0.003] and in the between-category comparison [*χ*^2^ (2, 100) = 9.36, *p* = 0.009] conditions, than in the within-category comparison which gave more consistent, texture-based, profiles. There was no significant difference between the no-comparison and the between-comparison conditions [*χ*^2^ (2, 100) = 3.33, *p* = 0.189]. In the highly distinctiveness condition, we found the same pattern of results: more shape consistent and more inconsistent profiles in the no-comparison [*χ*^2^ (2, 100) = 47.36, *p* < 0.001] and in the between-category comparison [*χ*^2^ (2, 100) = 35.01, *p* < 0.001] conditions, than in the within-category comparison. There was no significant difference between the no-comparison and the between-category conditions [*χ*^2^ (2, 100) = 1.71, *p* = 0.426].

#### 2.2.4 Control experiment: Are children able to use our 2D implementation of texture?

We also ran a control study with a 2D texture-only condition for both the high and low distinctiveness conditions to test whether our results could be explained in terms of texture accessibility differences across distinctiveness levels. To investigate this question, we used the same forced-choice paradigm as in Experiment 1. We first introduced a standard item with a given implementation of texture and shape. Then, we displayed two transfer items, one with the same texture but a different shape, the second differed on both texture and shape. Children who were able to detect texture similarities should choose the same-texture items. Otherwise, their choice between the two transfer items should random.

A total of others 60 preschoolers participated in this control experiment: 30 younger (16 female, mean age = 53.8 m ± 3.2, range: 48-59 m) and 30 older (16 female, mean age = 66.5 m ± 4.2, range: 60-71 m). They were all recruited with the same procedure as in Experiment 1 but did not participate in the two main experiments. The procedure and materials were identical to the 1–0 condition in Experiment 1 except that an unrelated item (i.e., with a different texture and a different shape) replaced the shape-match object. In both conditions, older (high: mean = 92.0 ± 22.4; low: mean = 97.3 ± 7.0) and younger children (high: mean = 81.3 ± 23.3; low: mean = 81.03 ± 29.7) selected the texture match in a vast majority of cases and performance was beyond texture choices in the best conditions in Experiment 1. This shows that performance in the low distinctiveness conditions cannot be explained by a failure to detect identical textures.

### 2.3 Discussion

This experiment assessed the role of stimulus distinctiveness and its interactions with comparison formats in a novel noun generalization task. Firstly, our findings align with the existing literature (Augier and Thibaut, 2013; Namy and Clepper, 2010) showing that within-category comparisons allowed children to find the unifying dimension, our implementation of texture, despite its lower *a priori* saliency. Secondly, our results underscore the limited impact of between-category comparisons, which did not significantly differ from the single stimulus condition. These two conditions confirmed former studies and prompted a majority of shape matches. The presence of a contrast object did not add much to the single condition (no-comparison). This is in line with some previous studies (Augier and Thibaut, 2013) and confirms Hammer (2015) analysis. Thirdly, while within-category comparisons gave more texture choices than between-category comparisons and no-comparison situations whatever the distinctiveness level, children selected the 2D texture match beyond chance only in the high-distinctiveness condition. These results support the hypothesis that less distinctive stimuli are more difficult to analyze and led to more random choices (see Cimpian and Markman, 2005; Tek et al., 2012). On the contrary, results are not compatible with the hypothesis that shape saliency would prevent 2D texture-based generalization.

So far, we have demonstrated that distinctiveness plays a role in the comparison paradigm. By construction, however, the two dimensions, shape and 2D texture had the same distinctiveness value, either high or low, in all the stimuli. With this paradigm, we cannot know whether children selected the 2D texture choice in the high-distinctiveness conditions because of a highly distinctive 2D texture which made it more salient, or because of a highly distinctive shape which made it easy to identify and to reject as irrelevant or both. To explore this question further, we manipulated shape and 2D texture distinctiveness independently in Experiment 2.

## 3 Experiment 2

Experiment 1 demonstrated that stimulus distinctiveness had a significant effect on 2D texture choices in our novel noun generalization task. Results showed that only within-category comparisons elicited a majority of 2D texture-based generalizations, independently of age. Between-category comparisons did not elicit 2D texture-based generalizations. For this reason, Experiment 2 focused exclusively on within-category comparison conditions. To obtain a deeper understanding of how each dimension distinctiveness influences the comparison process, separately, we systematically varied shape distinctiveness and 2D texture distinctiveness, resulting in four conditions:

- High-shape/high-texture (HS/HT) condition in which both shape and 2D texture were highly distinctive.
- High-shape/low-texture (HS/LT) condition in which shape was highly distinctive and 2D texture less distinctive.
- Low-shape/high-texture (LS/HT) condition in which 2D texture was highly distinctive and shape less distinctive.
- Low-shape/low-texture (LS/LT) condition in which both shape and 2D texture were less distinctive.

High-shape/high-texture (HS/HT) and low-shape/low-texture (LS/LT) correspond to the two conditions of distinctiveness that were compared in Experiment 1. Therefore, we expect to replicate our findings, which are more 2D texture-based responses with highly distinctive objects (HS/HT) than with low distinctive objects (LS/LT). For the two new conditions, in the high-shape/low-texture (HS/LT) condition, two opposing hypotheses can be considered: (1) a more distinctive shape might increase the saliency of shape, resulting in a greater number of same-shape choices, or (2) since the learning examples do not share the same shape, children might understand that shape can be disregarded as a potentially unifying property for generalization which would pave the way to the 2D texture dimension. In the low-shape/high-texture (LS/HT) condition, the analogous hypothesis can be made (1) a less distinctive shape might decrease shape saliency, resulting in a fewer number of shape choices, or (2) since shape is less salient, and because stimuli are hypothesized to be more difficult to parse into their constitutive dimensions, it might be more difficult to decide that shape can be disregarded as a potentially unifying property for generalization.

### 3.1 Method

#### 3.1.1 Participants

Two hundred and forty children participated in this study and were tested individually in a quiet room at their school. They were randomly assigned to one of the four experimental conditions with 60 children per condition. See Table 4 for population. The same ethical protocol as in Experiment 1 was followed.

**Table 4:** Participants’ age and gender for each condition of Experiment 2.

	Conditions				Comparison
Condition of distinctiveness	High-shape/low-texture	High-shape/high-texture	Low-shape/high-texture	Low-shape/low-texture	—
Number of participants	60	60	60	60	—
Age (in months)	61.96 ± 8.47	62.60 ± 7.96	62.51 ± 8.32	62.29 ± 7.57	*F* (3,236) = 0.07, *p* = 0.97
Gender (F/M)	37/23	30/30	44/16	30/30	—

F, females; M, males.

#### 3.1.2 Materials

The same high-and low distinctive shapes and textures as in Experiment 1 were used to create two new sets of four artificial grey-scale objects, one for each of the two novel conditions of distinctiveness, HS/LT and LS/HT. In the high-shape/low-texture (HS/LT) condition, highly distinctive shapes were associated with low distinctive textures and in the low-shape/high-texture (LS/HT), low distinctive shapes were associated with highly distinctive textures.

**Figure 4:**
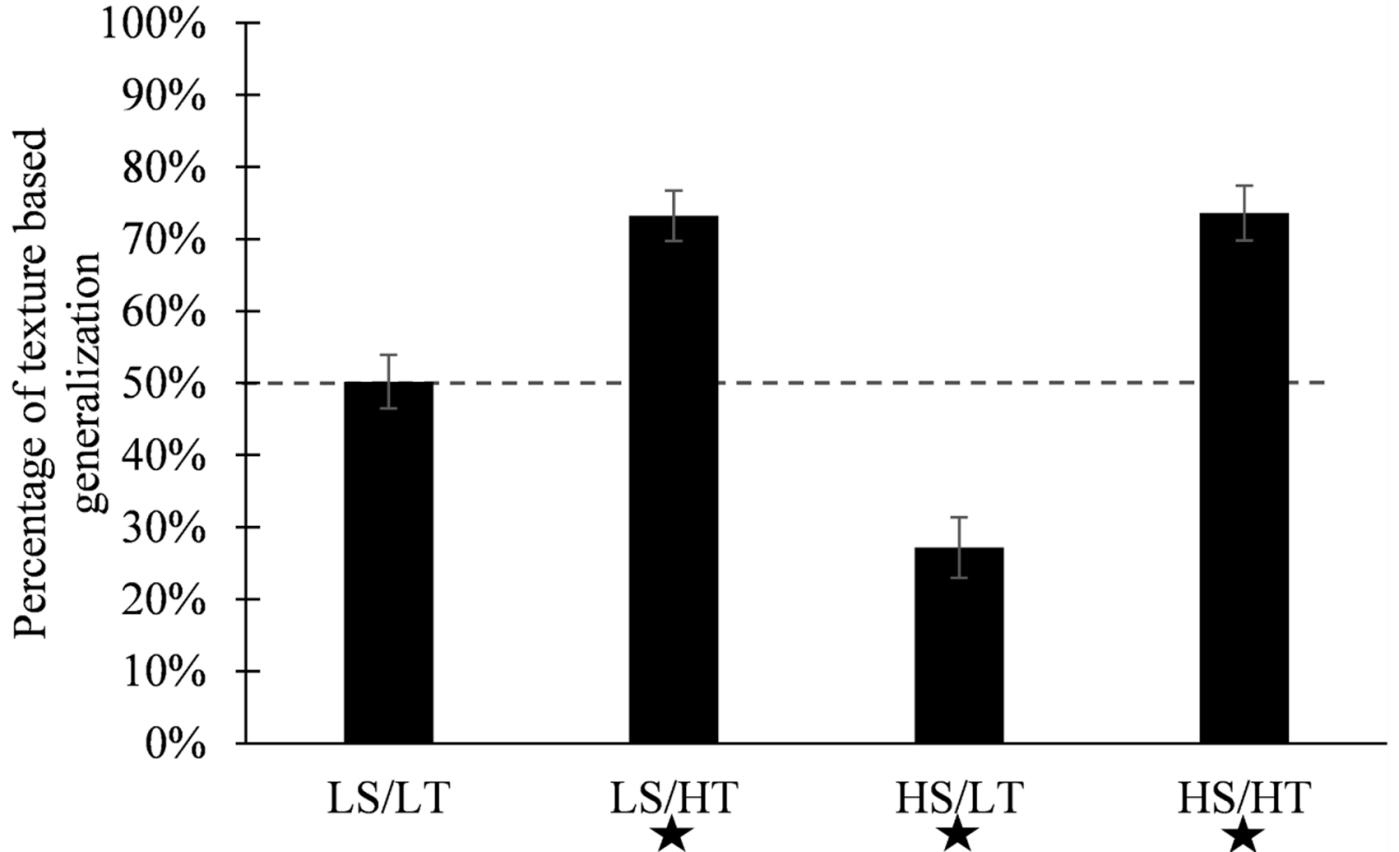
Mean percentage of texture-based categorization as a function of the condition of comparison and distinctiveness. LS/LT stands for low-shape/low-texture. LS/HT stands for low-shape/high-texture. HS/LT stands for high-shape/low-texture. HS/HT stands for high-shape/high-texture. A star at the bottom of a bar means that the corresponding condition significantly differed from chance (50%). Vertical bars represent standard errors of the mean. ^*^p < 0.05.

We used the same procedure as in Experiment 1 to assess stimulus distinctiveness. We asked 43 adults to rate the perceptual distinctiveness of pairs of objects on a 7-point Likert-like scale (ranging from not distinctive at all to extremely distinctive). Two ratings were carried out, one for texture distinctiveness the other for shape distinctiveness, for the stimuli of the low-shape/high-texture and high-shape/low-texture conditions and compared these scores to the ones we obtained in the two conditions from Experiment 1. Results confirmed that shapes were judged more distinctive in high-shape/low-texture and high-shape/high-texture than in low-shape/low-texture and in low-shape/high-texture (all *p* < 0.001, see Table 2). Textures were also judged more distinctive in high-shape/high-texture and low-shape/high-texture than in low-shape/low-texture and in high-shape/low-texture (all *p* < 0.001, see Table 2).

As in Experiment 1, the two learning stimuli (standards) had the same texture but different shapes. The two test objects were the same in the four conditions, a shape match and a texture match as in Experiment 1. We used the same pseudo-words as in Experiment 1.

#### 3.1.3 Procedure

The procedure was the same as the within-category comparison condition of Experiment 1.

#### 3.1.4 Data analysis

As for Experiment 1, we calculated for each participant the percentage of texture match choices. The first two trials were training trials and were excluded from the analysis. The data came from the five following trials. Thus, the scores ranged from 0 to 100%, with increments of 20%. Then, we computed the mean percentage of texture match choices of all participants in each of the four conditions.

#### 3.1.5 Statistical analysis

We conducted an ANCOVA with 4 distinctiveness (high-shape/high-texture vs. high-shape/low-texture vs. low-shape/high-texture vs. low-shape/low-texture) as between-subject factors and age as a covariate on the percentage of texture match choices. As in Experiment 1, we also compared the percentage of texture match choices with chance level (50%) and analyzed individual consistency patterns.

### 3.2 Results

#### 3.2.1 ANCOVA analysis

The ANCOVA revealed a significant effect of distinctiveness [*F* (3, 235) = 23.80, *p* < 0.001, *η*^2^_P_ = 0.23, BF_10_ >1,000, which corresponds to decisive evidence in favor of H_1_].

Post-hoc (see Figure 4) confirmed that there were more texture-based responses in the high-shape/high-texture condition (mean = 70.92%, IC = 61.57–80.26) than in the low-shape/low-texture condition (mean = 49.33%, IC = 40.77–57.90; *p* = 0.005, *d* = 0.62) and in the high-shape/low-texture condition (mean = 26.42%, IC = 17.85–34.98; *p* < 0.001, *d* = 1.27). There were also more texture based-responses in the low-shape/high-texture condition (mean = 73.33%, IC = 64.36–82.30) than in the low-shape/low-texture condition (*p* = 0.001, *d* = 0.68) and in the high-shape/low-texture condition (*p* < 0.001, *d* = 1.34). Finally, there were more texture based-responses in the low-shape/low-texture condition than in the high-shape/low-texture condition (*p* = 0.005, *d* = 0.62). However, there was no significant differences between the high-shape/high-texture condition and the low-shape/high-texture condition (*p* = 0.973, *d* = −0.43). Overall, the ANCOVA and the post-hoc indicate that (1) when texture was highly distinctive, children selected the texture match more often than in the low texture distinctiveness conditions, regardless of shape distinctiveness (2) in the low texture distinctiveness condition, children selected more frequently the shape match when the shape was distinctive. These results suggest that the distinctiveness of both dimensions was influential, high texture distinctiveness eliciting texture choice, low texture distinctiveness giving the lead to shape. This result has to be combined with the no-comparison condition in Experiment 1, showing the high shape distinctiveness would drive children’s generalization choices (see general discussion).

The ANCOVA also revealed a significant effect of age [*F* (1, 235) = 10.57, *p* = 0.001, *η*^2^_P_ = 0.04, BF_10_ = 14.82, which corresponds to strong evidence in favor of H_1_] suggesting an increase of texture-based responses with age.

#### 3.2.2 Comparison to chance (50%)

In each of the four experimental conditions, we conducted a comparison of the percentage of texture-based responses against chance (50%) using a one-sample *t*-test. Results confirmed that texture-based responses were significantly above chance (50%) in the two highly distinctive texture conditions (low-shape/high-texture: *p* < 0.001, *d* = 0.66; high-shape/high-texture: *p* < 0.001, *d* = 0.57). Texture-based responses were below chance (i.e., more shape-based responses) in the high-shape/low-texture condition (*p* < 0.001, *d* = −0.70). The percentage of texture-based responses in the low-shape/low-texture condition was not significantly different from chance (*p* = 0.879, *d* = −0.02).

#### 3.2.3 Consistency patterns analysis

Results were also confirmed by analyses of individual consistency patterns of answers (see Experiment 1): there were more texture-consistent profiles in the two high-distinctiveness texture conditions (low-shape/high-texture and high-shape/high-texture) than in the two other conditions (all *p* ≤ 0.001). As expected, there were also more inconsistent profiles in the low-shape/low-texture condition than in all the other conditions (all *p* ≤ 0.001), which suggests that participants were unable to parse the stimuli into their dimensions appropriately and thus answered randomly.

### 3.3 Discussion

Experiment 2 manipulated the distinctiveness of each dimension separately and systematically assessed the role of shape and texture in the frame of a novel noun generalization task with comparison. There were two important results. The first was that, texture-based options were selected significantly beyond chance when texture was distinctive, whatever the distinctiveness of shape. This suggests that the shape saliency can be overcome when a less salient dimension (here texture) is more distinctive. Secondly, a highly distinctive shape and a low distinctive texture led to a majority of shape-choices despite the strong evidence provided by the examples that shape was not the unifying feature. Thus, when selecting the less salient dimension (e.g., texture), the process is primarily driven by the intrinsic characteristics of that dimension (i.e., distinctiveness), rather than by the inhibition of the more salient dimension (here, shape). Indeed, if the *a priori* saliency of shape was the main issue, especially when shape is highly distinctive, children would never have access to texture, regardless of its level of distinctiveness.

## 4 General discussion

In two experiments, we assessed the role of dimension distinctiveness (high vs. low distinctiveness) on novel object nouns generalization, in three comparison formats. Building upon prior studies revealing that children often generalize a novel noun to objects sharing a salient dimension (e.g., a similar shape) (Imai and Haryu, 2004; Landau et al., 1988), we sought to identify conditions eliciting novel word generalization based on a priori less salient properties, be it comparison format or dimension distinctiveness. Consistent with several other studies (Augier and Thibaut, 2013; Gentner and Namy, 1999; Graham et al., 2010; Namy and Clepper, 2010; Namy and Gentner, 2002; Thibaut and Witt, 2023), we found that children gave texture-based generalizations of a novel object noun when two examples of the same target category were introduced, which was not the case with objects coming from different categories (between-category), or single training objects. As for our main question, stimulus distinctiveness had a significant effect on children’s selections and interacted with presentation formats.

In Experiment 1, in the no-comparison condition, a majority of children generalized the novel nouns to the shape-match confirming the importance of shape in children generalization (Kucker et al., 2019). Secondly, the between-category condition also elicited a majority of shape-based answers and did not differ from the no-comparison condition. Thirdly, the effect of within-category comparisons on texture selections was modulated by stimulus distinctiveness: only the highly distinctive stimuli gave a percentage of texture-based generalization beyond chance level.

Experiment 2 crossed shape and texture distinctiveness. The important result was that only the two highly distinctive texture conditions (high-shape/high-texture and low-shape/high-texture) elicited a majority of texture-based generalizations, independently of the two levels of shape distinctiveness.

### 4.1 The importance of shape in children’s word generalization

Our results, with unfamiliar stimuli confirm the importance of shape in the single (no-comparison) condition. This is consistent with what decades of research has shown and that has been interpreted as evidence for a shape bias in lexical learning or in terms of dimension saliency (Imai et al., 1994). In the no-comparison condition, the shape bias was stronger when both the shape and the texture were highly distinctive, which is consistent with the idea of a bias for shape. Since the texture was highly distinctive, the results in favor of shape cannot be only a byproduct of dimension saliency. Thus, the reference to shape is more pronounced when it is more distinctive, thus, according to our hypothesis, easier to parse as a separate dimension.

In the comparison conditions, what is new is that the preference for shape over texture was even more pronounced in the high-distinctiveness case. The lower percentage of shape-matches with low distinctiveness objects suggest either that they understood that shape was not relevant but failed to find another dimension and responded more randomly; or that they had more difficulties to parse the stimulus into its constituent dimensions and use them when they were less distinct. This latter view gains further support from the outcomes of the low-shape/low-texture condition (LS/LT), which resulted in chance-level responses. Note that the structure of stimuli also had an effect in Cimpian and Markman (2005), but in a different way. The authors showed that the shape bias was reduced with stimuli displaying a complex shape (e.g., a bicycle, a flower) rather than a simple shape (cookie, apple). Even though the interpretation has not been undisputed (see Smith and Samuelson, 2006), this effect also suggests an effect of stimulus structure, which might go beyond shape itself as advocated by Smith and Samuelson (2006) in the case of familiar objects. In the current study, this modulation of the role of shape by stimulus structure extends to unfamiliar stimuli. Less distinctive stimuli led to a weaker shape influence because, even though the dimensions could be identified in the low-distinctiveness case (see our control experiment), it is more difficult to manipulate them, as suggested above. Although Cimpian and Markman (2005) controlled for shape similarity, their data suggest that shape becomes a less reliable basis for generalization when stimuli consist of a larger number of functionally distinct parts. This result indicates that shape is generally easy to detect as a dimension. However, when shape is less distinct or constructed around numerous functional subparts—making the shape of the stimulus less salient—participants are less likely to focus on them, a phenomenon that Regehr and Brooks (1993) referred to as “holistic individuation.”

### 4.2 How distinctiveness interacts with comparison

One key question of this paper was the role distinctiveness would play. Our experiments confirmed our main hypothesis, that is that stimulus distinctiveness influences children’s choices and interacts with the comparison format. With 2D unfamiliar stimuli, our results show that it is not the influence of shape *per se* or the comparison format *per se* but how these factors, the influence of which is well-known, interact with stimulus distinctiveness. First, in the within-category comparison condition, texture became the dominant choice only in the high-distinctiveness texture condition, not in the low-distinctiveness case. Second, this result was independent of shape distinctiveness. Third, the prevalence of shape choices in the high-shape/low-texture shows how shape distinctiveness interacts with texture distinctiveness. Finally, shape plays a role when texture (the relevant dimension) is less distinctive, as shown by the higher number of texture-based responses in the low-shape/low-texture than in the high-shape/low-texture condition. However, in the low-shape/low-texture condition, children were at chance, suggesting that they had difficulty consistently selecting a dimension, likely because they had difficulty parsing the stimuli into their constitutive dimensions.

How does shape distinctiveness play its role as a function of comparison status? In fact, in Experiment 1 it went in opposite directions in the no-comparison and the within-category conditions. Indeed, in the former case, children selected more often the shape match in the high distinctiveness condition than in the low distinctiveness condition, whereas in the within-category comparisons, the high shape distinctiveness condition gave more texture-based responses (than with low shape distinctiveness condition). However, Experiment 2 revealed that shape distinctiveness was in fact less decisive than texture distinctiveness.

How does texture distinctiveness play its role in the within-category condition? Based on Hammer et al. (2008, 2009) analysis (see introduction), in the within-category comparison conditions similarities between the examples should be considered relevant whereas differences should be discarded as irrelevant. Given that the two learning examples did not have the same shape, this feature could be considered as irrelevant and a common texture increased its conceptual relevance. Experiment 2 tested whether distinctive shapes are easier to ignore or more distinctive textures are easier to parse. Results were in favor of the latter. The absence of difference between the high-shape/high-texture and the low-shape/high-texture shows that discarding shape is less important than detecting common textures, which is more easily done when *a priori* less salient dimensions are distinctive. As shown by our control experiment, the lower percentage of texture selections in the less distinctive texture case was not due to difficulties perceiving texture similarities.

### 4.3 No role for between-category comparisons?

One additional purpose of the present paper was to assess the role of between-category comparisons. Hammer (2015) and Hammer et al. (2008, 2009) argued that between-category similarities can be discarded as relevant whereas between-category differences are potentially relevant for generalization. Our results show that children did not benefit from these between-category comparisons. This result is consistent with previous findings by Augier and Thibaut (2013) and Namy and Clepper (2010) who also found limited evidence that children used the non-diagnosticity of shape in between-category conditions (same shape for the stimulus from the two categories) as this condition did not elicit selections of the texture match, in both distinctiveness conditions.

Price and Sandhofer (2021) also observed that between-category comparisons had less influence on children’s generalization of novel nouns when shape was shared by the stimuli. As previously discussed, note that a dimension which is shared by two categories (between-category commonality) is not, in itself, irrelevant for these categories. While such a shared dimension cannot be used to distinguish the two categories in the targeted context, we concur that it may still be important for later superordinate level categorizations. However, we mean that a between-category common dimension does not contribute to define each category as a specific set of objects, or differentiate them. With respect to adjectives, Ankowski et al. (2013) proposed that the category structure might influence the impact of within-and between-category comparisons. Specifically, they argued that high-density categories (i.e., categories in which members share many common features relevant to category membership, with minimal variation in irrelevant features) might benefit more from between-category comparisons (referred to as “contrast” in their study), while low-density categories might benefit more from within-category comparisons. In our study, low-distinctiveness categories might correspond to their low-density categories, while high-distinctiveness categories could correspond to their “high-density” categories. However, our results do not align with those of Ankowski et al. (2013), as in our low-distinctiveness condition, between-category comparison did not facilitate word generalization. However, they worked on adjective generalization, rather than on noun generalization. It could be that finding to property of an object an adjective refers to is easier to find when two similar objects (the objects are stable) differ on one property: adjectives would stress (“this one is daxy, this one is not daxy”) the difference between two otherwise similar objects (see also Waxman and Klibanoff, 2000).

### 4.4 Distinctiveness, novel noun learning, and levels of categorization

Finally, we believe that our results have general consequences for category learning and naming in the real world. Indeed, even though the experiments were not designed to test this hypothesis, stimulus distinctiveness could explain part of the well-established difference between the basic and the subordinate levels of categories. In the concept literature, ever since Rosch et al. (1976) seminal work, the difference between levels of categorization have been mainly conceived and described in terms of shared features. Basic level categories were defined as categories for which category members have both a high number of features in common (e.g., all tables share many features) and a high number of features that differentiate them from members of contrast categories (e.g., tables and cupboards differ along many features). For these reasons, they are more distinctive than subordinate categories (Murphy, 2002). Indeed, members of subordinate categories have many common features, but have fewer features differentiating them from members of contrast subordinate categories (e.g., green apples and Cox apples have many features in common).

Beyond this classical description in terms of shared features, we want to argue that the basic and the subordinate level categories could also be described in terms of feature distinctiveness as defined here. Thus, according to this description, both levels might be built around the same set of features but would differ in feature distinctiveness: features would be less distinctive in the subordinate categories than in the basic-level categories. For example, two subordinate categories like a poodle and a spaniel are built, to a large extent, around the same set of structural features (types of hairs, types of head, types of tail, type of sounds, etc.). However, the instantiations of their constitutive features are not very distinctive. At the basic level, two categories such as dogs and cats can also be described along the same sets of features. From our perspective, these two basic-level categories share a set of common features, albeit these features would be more distinctive (as we define it here) than those that characterize subordinate-level categories. For instance, the vocalizations of dogs and cats exhibit significant differences, whereas the distinctions in sound types between Poodles and Scottish Terriers might be subtler. Similarly, the characteristics of dogs’ and cats’ fur show more noticeable disparities compared to the differences in fur between Poodles and Scottish Terriers. In this framework, our data might provide a new, complementary, interpretation of the basic level advantage in learning. More distinctive stimuli (easier to parse into their constituents) would, in our view, define basic level categories, give more clear-cut and more accurate generalization patterns of categorization than less distinctive stimuli which would characterize subordinate level categories. This view would also explain why basic-level categories are the first to be learnt, before subordinate categories (see Markman, 1989; Murphy, 2016; Rosch et al., 1976). Indeed, basic level categories, being built around more distinctive features than subordinate level categories, would be easier to master than the latter. This higher distinctiveness of the basic level categories would help children to parse the stimuli into their components more easily. Of course, endorsing this view or not is not necessary to follow the aims and results of the present paper.

### 4.5 Limitations

These findings regarding the influence of distinctiveness on children’s generalization were obtained within a specific experimental context. Notably, we used materials distinct from real-life objects, with variations limited to shape and texture while maintaining uniform size and color across items, thus with simpler, less realistic objects compared to everyday objects. Furthermore, it is essential to recognize that our findings may not necessarily generalize beyond the specific context of simultaneous comparison tasks. In real-world situations, children may encounter isolated objects and/or mentally compare them to stored representations (i.e., memory). The interaction with distinctiveness might differ, particularly because sequential comparison inherently engages working memory processes (Lawson, 2017). A recent study (Hartley and Whiteley, 2024) provides results that seem to be consistent with ours. With similar materials (pictures of unfamiliar objects varying in shape and texture), however, the authors show with two-and three-year-old children, that within-category comparisons enhance generalization and retention compared to a unique example. Their results show that in the comparison condition, young children extended labels to referents from memory.

## 5 Conclusion

In conclusion, our findings confirm that distinctiveness plays a key role in within-category comparisons during novel word generalization. Specifically, we showed that less distinctive stimuli are more challenging for children to interpret accurately, while highly distinctive stimuli facilitate generalization. Importantly, our results suggest that children are more likely to generalize a novel name based on a very dimension. When children selected shape as the matching dimension, even though it did not characterize both stimuli, this may reflect difficulty in identifying a more relevant dimension. This choice of shape could reflect either (1) an inability to inhibit the salience of shape or (2) difficulty in flexibly encoding the stimuli based on an alternative dimension. Our findings are consistent with recent evidence that cognitive flexibility, rather than inhibition or working memory, is a better predictor of children’s performance on generalization tasks (Lagarrigue and Thibaut, 2020, 2025).

## Data Availability

The datasets presented in this study can be found in online repositories. This data can be found here: https://osf.io/8uw9h/?view_only=50746e429e8344c78c6fe5dd5d11aed3.
